# Non-plasmonic nanoantennas for surface enhanced spectroscopies with ultra-low heat conversion

**DOI:** 10.1038/ncomms8915

**Published:** 2015-08-04

**Authors:** Martín Caldarola, Pablo Albella, Emiliano Cortés, Mohsen Rahmani, Tyler Roschuk, Gustavo Grinblat, Rupert F. Oulton, Andrea V. Bragas, Stefan A. Maier

**Affiliations:** 1 Laboratorio de Electrónica Cuántica, Departamento de Física, FCEN, Universidad de Buenos Aires, Intendente Güiraldes 2160, C1428EGA Buenos Aires, Argentina – IFIBA CONICET, Argentina; 2 The Blackett Laboratory, Department of Physics, Imperial College London, London SW7 2AZ, UK

## Abstract

Nanoplasmonics has recently revolutionized our ability to control light on the nanoscale. Using metallic nanostructures with tailored shapes, it is possible to efficiently focus light into nanoscale field ‘hot spots'. High field enhancement factors have been achieved in such optical nanoantennas, enabling transformative science in the areas of single molecule interactions, highly enhanced nonlinearities and nanoscale waveguiding. Unfortunately, these large enhancements come at the price of high optical losses due to absorption in the metal, severely limiting real-world applications. Via the realization of a novel nanophotonic platform based on dielectric nanostructures to form efficient nanoantennas with ultra-low light-into-heat conversion, here we demonstrate an approach that overcomes these limitations. We show that dimer-like silicon-based single nanoantennas produce both high surface enhanced fluorescence and surface enhanced Raman scattering, while at the same time generating a negligible temperature increase in their hot spots and surrounding environments.

## Abstract (web summary)

Metallic nanoantennas can enhance and confine electromagnetic fields, however, localized heating hinders many applications. Here, Caldarola *et al.* demonstrate both high near-field enhancement and ultra-low heat conversion in the visible-near infrared region using silicon dimer nanoantennas with 20 nm gaps.

## Graphical abstract

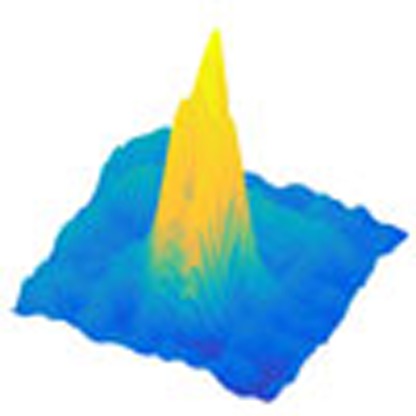

Metallic nanoantennas have been widely used to efficiently enhance electromagnetic fields and confine them to nanometric volumes, particularly at optical frequencies^1^. This local enhancement is due to the resonant excitation of surface plasmons, collective oscillations of the conducting electrons in metals^2^. Enhanced light-matter interactions mediated by plasmons enable many applications, such as information processing^3^, ultrasensitive (bio)detection^4^, surface enhanced Raman scattering (SERS)^5^,^6^, surface enhanced fluorescence (SEF)^7^ and nonlinear spectroscopy^8^,^9^,^10^. Gold and silver are commonly used in plasmonic devices because of their high DC conductivities^11^,^12^. However, at optical frequencies interband transitions play a detrimental role in these metals^13^. Losses arising from interband transitions occur when a valence electron in the metal absorbs a photon to jump to the Fermi surface or when an electron near the Fermi surface absorbs a photon to jump to the next unoccupied state in the conduction band^14^,^15^. This loss mechanism leads to Joule heating of the structure and its local environment^16^. In recent years several applications, such as photothermal cancer therapy^17^, photothermal imaging^18^, catalytic process enhancement in photocatalysis^19^ and photohermal biosensing^20^ have been proposed to take advantage of this nanoscale-generated (that is, highly localized) heat in metallic-based plasmonic nanoantennas.

For many other applications, however, localized heating is extremely detrimental for both the application/experiment and for the nanoantennas themselves^21^,^22^. Local heating of the plasmonic structures changes their refractive indices via the thermo-optic effect. Depending on the illuminating power, nanoantennas can even be reshaped and/or melted, thus strongly affecting their nanoscale lighting and photonic modulation capabilities^23^. Moreover, heat generated by the nanoantennas can vapourize the surrounding liquid/solvent media^24^ or affect nanoemitters, molecules and proteins close to them^25^. To circumvent the issues of losses and heating, the use of non-plasmonic materials, such as silicon, germanium or gallium phosphide (high refractive index dielectrics), has been theoretically proposed for the fabrication of nanoantennas with the ability to confine and enhance near and far-field light^26^,^27^. The magneto-optical response^28^,^29^,^30^,^31^,^32^ and directional light scattering^33^,^34^,^35^ of these materials have been explored and recent experimental efforts in this field have been devoted to controllably obtaining single spherical Si nanoparticles^36^.

In this work, we show experimental results that illustrate both high near-field enhancement (electromagnetic hot spots) and ultra-low heat conversion in the visible-near infrared (vis-NIR) region using Si dimer nanoantennas with 20-nm gaps. These hot spots are able to enhance the Raman scattering from a polymer thin film by up to 10^3^ times. Moreover, we report surface enhanced fluorescence of the same order from emitters in the vicinity of these non-plasmonic antennas. Finally, using molecular thermometry we compare the temperature increase in the gap and surrounding areas of Si dimers against Au-reference nanoantennas under the same experimental conditions, when illuminating at their resonance wavelength. We find Si dimer related heating to be 1/18 times that of the Au plasmon-analogue system; with the last only doubling the field enhancement capability of the dielectric. Theoretical simulations are used to better understand and support these findings. Non-plasmonic nanoantennas open a new approach for surface-enhanced spectroscopies with the peculiarity of not perturbing the response of the target under evaluation by undesired local heating.

## Results

### Silicon nanoantennas design and enhancement

**Figure 1:**
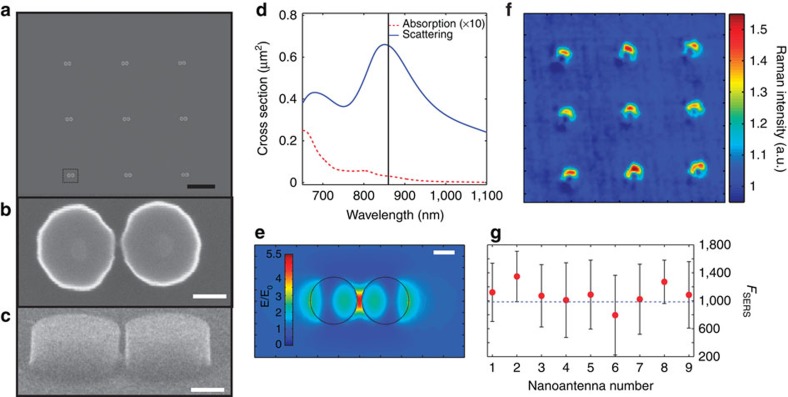
Design and performance of silicon nanoantennas. (**a**) SEM image of the array of nanostructures fabricated in Si on a silicon-on-insulator substrate. Each nanoantenna consists of two identical disks with a diameter of 220 nm, a height of 200 nm and a 20-nm gap in between. Scale bar, 2 μm. (**b**) SEM top-view and (**c**) lateral-view images of a single nanoantenna, indicated in the rectangle in **a**. Scale bar, 100 nm. (**d**) Numerical calculation results showing a scattering resonance at *λ*=860 nm (vertical black line) and a low absorption cross section for the Si dimer in PMMA. Note that the absorption curve is multiplied by a factor of 10. (**e**) Near-field distribution map for the silicon structure excited at resonance, showing good confinement of the electric field in the gap. Note that the maximum enhancement value is 5.5. Scale bar, 100 nm. (**f**) Experimental 2D normalized Raman map, showing enhanced signal coming from the molecules close to the nanoantennas. (**g**) SERS enhancement factors obtained for each individual nanoantenna shown in **f**, after volume normalization (see Methods, SERS mapping section, for details). The error bars show half the difference between the minimum and the maximum Raman intensity value in each nanoantenna. The dashed line corresponds to (*E*_max_*/E*_0_)^4^, from **e**.

Guided by numerical calculations (described in the Methods section, numerical calculations), we designed and then fabricated silicon dimer antenna arrays to evaluate their performance as optical nanoantennas in the near infrared region of the spectrum expecting, as predicted theoretically, good enhancement response in the near and far field together with ultra-low heat conversion. Figure 1a shows a scanning electron microscope (SEM) image of the fabricated arrays, while Fig. 1b,c exhibit the diameter, height and shape of a single Si-dimer nanoantenna with a 20-nm gap between the two disks; a very challenging configuration for this material. The fabrication methodology can be found in the Methods section, sample fabrication and preparation, while further characterization can be found in Supplementary Fig. 1 and Supplementary Note 1. These nanostructures present a broad scattering resonance in the NIR, whereas at the same time, the low-energy band gap of Si (∼1.1 eV), leads to a very low absorption in this region as shown in Fig. 1d. Figure 1e illustrates the calculated near-field distribution around a silicon dimer excited at resonance (*λ*=860 nm), describing the optical antenna performance. As this is a dipolar resonance of the structure, the maximum field enhancement occurs in the gap, reaching values close to 5.5 for *E/E*_0_. We note that theoretical simulations predict (*E/E*_0_)^4^ enhancements as large as ∼10^6^ (*E/E*_0_=∼32) for Si dimers with gaps as small as 4 nm (ref. 26), which would allow in principle single-molecule SERS detection^37^ (see Supplementary Fig. 2 and Supplementary Note 2). For comparison, we observe that gold structures such as bow–tie nanoantennas with sharp edges and 4-nm gap, have been shown to produce *E/E*_0_ values up to ∼40 (ref. 38), whereas Au disk dimers with similar inter-disk spacing distance to that of our Si dimers (20 nm) show values for the field enhancement which are higher by a factor of 2 (ref. 39). It is important to mention that the physical mechanism behind dielectric or metallic nanoantennas is essentially different. While dielectric antennas rely on the fields and displacement currents induced by the external electromagnetic radiation, in metallic ones their properties are based on the oscillations of the free-electron plasma^26^.

To experimentally probe the optical antenna performance (that is, the local field enhancement), a 200-nm thick poly(methyl methacrylate) (PMMA) polymeric film was deposited on top of the Si-nanoantenna sample to study the SERS effect. Using a home-built confocal microscope fed with a CW Ti:Sapphire laser, two-dimensional (2D) Raman maps were recorded by scanning the sample and sending the scattered light through an appropriate set of filters and into an avalanche photodiode (APD). To verify that there was adequate rejection of the laser line, the scattered light was sent to a spectrometer and Raman spectra were acquired at several selected points on the sample. For details refer to the sample fabrication and preparation and SERS mapping sections in Methods.

An image of the SERS signal from a PMMA film taken at the antenna resonance wavelength (*λ*=860 nm) is shown in Fig. 1f. To produce this image, the Raman image at *λ*=860 nm has been normalized with the scattering image taken at *λ*=890 nm. The normalized Raman map is quantified in the colour bar (see Methods, SERS mapping section, for a more detailed explanation). Finally, in Fig. 1g we calculate the SERS enhancement factor, *F*_*SERS*_ (see Methods, SERS Mapping section for details), for each antenna and compare it with the expected value, the fourth power of the peak^6^,^40^,^41^ of the computed field distribution shown in Fig. 1e (dashed line). As can be seen in, the *F*_*SERS*_ value computed for each single nanoantenna is around 10^3^, in good agreement with the expected value.

These results demonstrate that these dielectric structures do actually behave as nanoantennas, and that they are capable of enhancing electromagnetic fields confined into nanometric-sized hot spots^32^. Moreover, they could be used as an efficient platform for SERS experiments. For instance, silicon nanoantennas can be easily modified by silane molecules due to the ultrathin native oxide layer (see Supplementary Fig. 1 and Supplementary Note 1 for further details).

We turn now to consider in detail, the thermal behaviour of these antennas, trying to demonstrate experimentally that they produce ultra-low heating when illuminated with optical fields, in contrast to traditional plasmonic nanoantennas.

### Thermal mapping method

**Figure 2:**
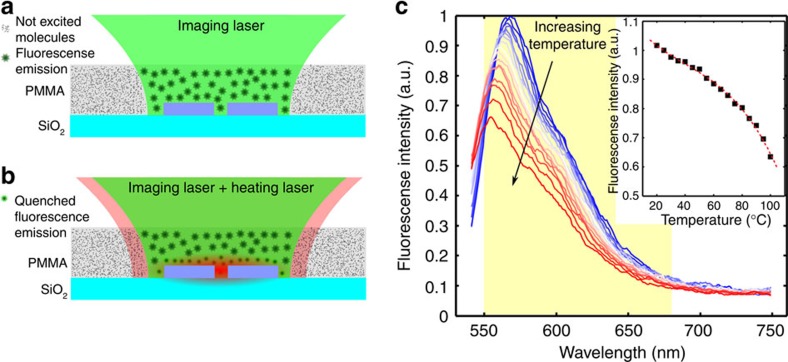
Molecular thermometry experiment. (**a**) Scheme for the fluorescence imaging experiments in the nanoantenna sample. The imaging laser excites Nile Red molecules embedded in the PMMA thin-film layer on top of the nanoantennas within the confocal spot. (**b**) When the heating laser is turned on, the nanoantennas increase their temperature and the molecules close to the nanoantennas decrease their fluorescence emission, resulting in a lower fluorescence signal. (**c**) Nile Red emission spectra taken at different temperatures. The marked zone shows the detection spectral range (550–680 nm). The inset shows the integrated intensity in the detection spectral range as well as the fit used as a calibration curve to extract the corresponding temperature values.

We used a thermal imaging method that combines both diffraction-limited spatial resolution (close to 370 nm) and molecular thermometry to evaluate the temperature behaviour of the nanoantennas. Temperature mapping is achieved by imaging the emission of the fluorophore Nile Red (7-diethylamino-3,4-benzophenoxazine-2-ona) with a home-made dual-beam confocal microscope (see Methods, Dual-beam confocal microscopy and imaging section for more details). Nile Red is a fluorophore with high quantum efficiency and photostability and is widely used as a polarity-responsive fluorescent molecule in many environments, including polymeric films^42^. Figure 2a,b schematically illustrate the temperature measurement principle and experiment. Samples are covered with a PMMA thin-film doped with Nile Red molecules, which act as thermal nanoprobes. A low power CW 532-nm imaging laser is used for molecular excitation of the Nile Red molecules, and the fluorescence emission is detected in the spectral range 550–680 nm. Recording images of Nile Red fluorescence serves dual purposes; on one hand it serves as a mapping tool, since the presence of the nanoantennas modifies Nile Red fluorescence emission, allowing access to the location of the nanoantennas. On the other hand, it probes local temperature changes since the Nile Red fluorescence profile is particularly sensitive to temperature changes, as shown in Fig. 2c. When the heating laser (CW Ti:Sapphire) is turned on, absorption in the nanoantennas produces a temperature increase of the surroundings which is sensed by the Nile Red molecules on-site. Over the measurement time, the whole structure thermalizes (represented schematically in Fig. 2b as dim red over the structure) and a reduction in the fluorescence emission from the molecules around the nanoantenna takes place (represented in the same Figure as a reduction in the size of the dots/stars).

This reduction in the molecular fluorescence is due to the thermal activation of non-radiative channels, as well as a shift of the spectrum to higher energies (see Fig. 2c). The latter effect is called thermochromism, and it is explained as a decrement of the environmental polarity with the temperature rise^43^. These combined effects give our intensity-based method a sensitivity of around 0.5% per °C, which is comparable to other polymer-dye based methods^44^,^45^,^46^. Although fluorescence intensity quenching measurements are straightforward, the emission intensity also depends on the dye concentration and the illumination intensity. Therefore, we have done a very careful initial calibration combined with several control experiments in order to identify and avoid artifacts (in Methods, see temperature calibration and thermal imaging section for details). Using the calibration in the inset of Fig. 2c, a temperature map is obtained from a fluorescence image, where each value represents an average temperature within the imaging laser spot. This procedure is repeated for several values of heating laser intensity, *I*_h,_ to vary the amount of local heating and trace changes in temperature.

**Figure 3:**
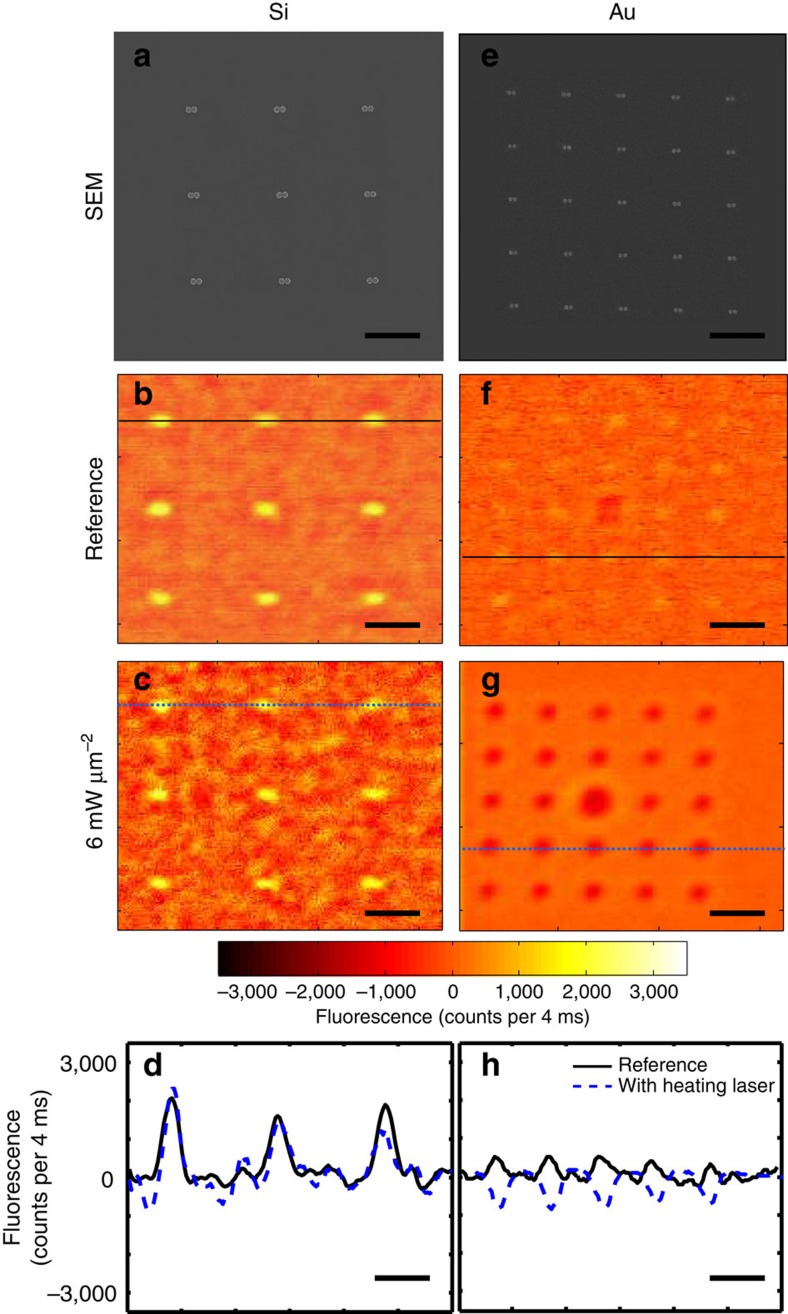
Nanoantenna fluorescence images. The top panels show SEM images of the nanoantenna arrays for reference, (**a**) for Si and (**e**) for Au. The four images below are fluorescence images corresponding to Si (left) and Au (right). Scale bar, 2 μm. Reference panels (**b**) and (**f**): fluorescence images without the heating laser turned on. (**b**) Silicon nanoantennas produce enhanced fluorescence while gold antennas (**f**) slightly enhance the fluorescence emission (see Supplementary Fig. 4 and Supplementary Note 4 for explanation). (**c**) and (**g**): Fluorescence images with the heating laser on at 6 mW μm^−2^. The color scale bar is the same for all the fluorescence images. Bottom panels: Intensity profiles taken from the rows indicated as lines in the fluorescence images. The intensity clearly drops for the gold antennas (**h**) but remains nearly constant for the silicon ones (**d**). The Nile Red molecules around the gold antennas are affected by drastic changes in temperature, leading to the observed intensity drop. In all the fluorescence images the background average value has been subtracted for better displaying.

To demonstrate the improved thermal performance of our dielectric nanoantennas when compared with traditional plasmonic nanoantennas, we also fabricated arrays of Au disk dimers with a dipolar resonance at *λ*=860 nm (see Supplementary Fig. 3 and Supplementary Note 3 for optical characteristics of the Au antennas). SEM images of the Si and Au arrays are shown in Fig. 3a,e, respectively. Confocal fluorescence imaging was performed on both sets of nanoantennas first without the heating laser, as shown in Fig. 3b for Si and Fig. 3f for Au, to be used as a reference for the assignment of the room temperature (*T*_r_=25 °C) fluorescence intensity across the maps. The imaging laser power was set three orders of magnitude lower than the heating laser power so that heating by the imaging laser can be considered negligible in both types of nanoantennas (in Methods, see Dual beam confocal microscopy and imaging section for details). Remarkably enough, the reference fluorescence images (Fig. 3b for Si and Fig. 3f for Au) have different behaviour over the nanoantennas. It is seen in these images that the silicon nanoantennas enhance the fluorescence emission with a contrast of 0 to 2,000 counts per 4 ms, while for the gold nanoantennas the contrast is significantly lower (0–500 counts per 4 ms). This is fully understood, and constitutes an additional striking advantage of the dielectric over the metal nanoantennas and is discussed in detail in the last section of this Article.

We then performed the heating experiment by simultaneously illuminating an array with both the imaging and the heating lasers (linear and circular polarization in the sample, respectively). Sequential images are taken for different heating laser intensities, *I*_h_. Fig. 3c,g show the fluorescence images attained with *I*_h_=6 mW μm^−2^ for silicon and gold antennas, respectively. To compare the fluorescence intensity with and without heating, Fig. 3d,h show the intensity line profiles with and without heating from the indicated rows of the nanoantenna arrays in the silicon and gold fluorescence images, respectively. It is easy to see that while the intensity profile remains nearly the same for the Si nanoantennas with and without the heating laser; that of the gold sample shows a pronounced intensity decrease around the antennas when the heating laser is turned on. This difference is due to the temperature increase around the Au antennas that can be transformed into a temperature value using the calibration curve in the inset of Fig. 2c.

### Temperature in silicon and gold nanoantennas

The local temperature around both Si and Au nanoantennas was studied as a function of the heating laser intensity. It should be noted that our experimental method gives an average temperature value, *T*, because in the confocal detection volume there are some molecules that are located in the heated zone and some other molecules that are not (see Fig. 2b).

**Figure 4:**
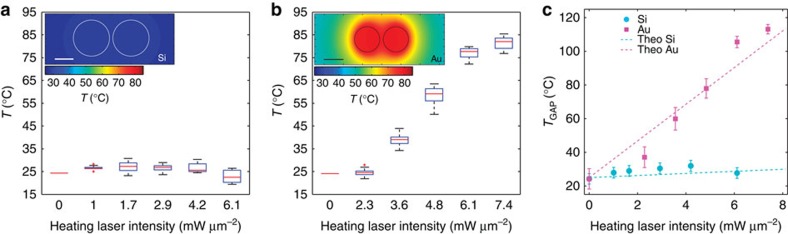
Temperature measurement in nanoantennas. Box plot for the average temperature *T*, measured for (**a**) silicon and (**b**) gold nanoantennas, shown in Fig. 3, excited at resonance. It can be seen that the Au nanoantennas significantly increase their temperature when *I*_h_ increases while the Si temperature remains nearly constant. The inset in each figure shows the calculated temperature map around the disks for *I*_h_=5 mW μm^−2^ in both cases. Scale bar, 100 nm. (**c**) Extracted temperature in the gap for selected silicon (cyan) and gold (magenta) nanoantennas as a function of the heating laser intensity at 860 nm. The dashed lines show the numerical calculations for the temperature at the gap, presenting good agreement with the experimental data. The error bars show the s.d. of the temperature measurements, obtained from error propagation from the fluorescence measurements.

The measured average temperature is shown as box plots in Fig. 4 for silicon (Fig. 4a) and gold (Fig. 4b). The horizontal axis shows the intensity of the heating laser for the polarization parallel to the nanoantennas, since this component is resonant with the antennas and therefore heats the structures. For each box, the central red mark is the median of the array of 9 (25) Si (Au) nanoantennas on the sample; the edges of the box are the 25th and 75th percentiles, and the whiskers extend to the most extreme data points that are not yet considered outliers (points are drawn as outliers if they are larger than *q*_3_+*1.5*(*q*_3_−*q*_1_) or smaller than *q*_1_−*1.5*(*q*_3_−*q*_1_), where *q*_1_ and *q*_3_ are the 25th and 75th percentiles, respectively). These outliers may be due to defects in the fabrication of few of the antennas. These results clearly evidence that the environment around the silicon nanoantennas remains nearly constant in temperature, while that of the gold nanoantennas increases by >60 °C (in the range of powers we used), demonstrating that dielectric nanoantennas produce local enhancement with minimal heating. In the inset of each figure a calculated temperature map was added for the case of 5 mW μm^−2^ heating laser intensity. Note that the highest temperature zone is confined over the nanoantenna including the gap, where the temperature is, for the Au case, ∼30 °C greater than in a region near the border of the map. A similar thermal behaviour can be found for the Si antennas at higher powers (see Supplementary Fig. 5 and Supplementary Note 5). This spatial dependence must be taken into account to obtain the temperature in the gap, which is the relevant temperature for SERS experiments, since the molecules sense the local electric field and the highest contribution comes from the hot spot in this gap.

To obtain the temperature in the gap of the nanoantennas (*T*_GAP_), we integrated the numerically calculated three-dimensional (3D) temperature distribution for each heating laser intensity (a 2D map at a fixed *z*-value of this distribution is shown in the insets of Fig. 4). From these calculations we obtain a proportionality factor relating the measured average temperature with the temperature in the gap (see Supplementary Fig. 6 and Supplementary Note 6 for details). This factor was then applied to the experimentally measured average temperature to calculate a temperature value in the gap. Figure 4c shows the gap temperature as a function of the heating laser power. As expected, the temperature increase in the gap is ultra low for the Si nanoantennas, while it exceeds 80 °C for the gold nanoantennas. This fact is in accordance with theoretical predictions, and a good agreement between these extracted gap temperatures and those extracted from numerical calculations (dashed lines) was found. From Fig. 4c we calculated a ratio between heating slopes (Au/Si) of 17.6. In addition, it is worth mentioning that numerical calculations of the temperature increase for heating laser intensities up to 120 mW μm^−2^ show that the gold nanoantennas would raise their temperature by >1,200 °C, while for the Si nanoantennas the temperature increment would not exceed 100 °C. Remarkably, by increasing less than five times the incident power for the Si nanoantennas we can get the same Raman signal than for the Au-analogue but with 75% less heating (refer to Supplementary Note 5 for detailed calculation). This substantial difference in the thermal behaviour for the two types of nanoantennas would allow working with much higher incident fields for the dielectric case, which could serve, for example, to improve the efficiency of light frequency up-conversion in nonlinear applications^8^,^9^. We note here, that the above mentioned value of 75% corresponds to processes which scale with (*E/E*_0_)^4^. For lower order processes this factor would be even more favourable for the dielectric nanoantenna.

To sum up, in this section we demonstrated both experimentally and theoretically that silicon nanoantennas excited at resonance do not heat appreciably while the gold nanoantennas heat significantly, even at low heating laser intensities.

### Surface enhanced fluorescence

In addition to the Raman scattering enhancement already shown in Fig. 1f,g, we also measured a remarkable Nile Red fluorescence enhancement generated by the Si-nanoantennas, as seen in Fig. 3b, in contrast to the dim fluorescence produced by this particular set of Au-nanoantennas (Fig. 3f). This behaviour is fully in accordance with the predictions of the numerical calculations where an ideal dipole is placed at the gap of each type of antenna and the radiative and non-radiative contributions of its emission are calculated over a broad spectral range^26^. Even though the increased local field intensity results in an enhanced molecular absorption rate for both kinds of nanoantennas, the molecular emission process is very different in nature for the gold and silicon cases. In general, fluorescence enhancement depends on two factors: the coupling of the emission to the radiative dipole mode of the nanoantenna, and the radiative efficiency of the dipole mode itself. Competing with this, coupling to non-radiative modes contributes to fluorescence quenching. However, since the continuum of quenching modes arises through the imaginary part of the permittivity (present in metals), having an almost real dielectric constant for the Si resonator supresses the quenching effect, leading to the observed behaviour (see Supplementary Fig. 4 and Supplementary Note 4 for details). Anger *et al.*^47^ very nicely illustrate limitations of metal particles in SEF due to the quenching phenomena. For metals, a spacer layer between the antenna and the emitter is needed to diminish quenching and achieve stronger fluorescence (both for antennas on and off resonance with the absorption or emission windows of a particular emitter), but as a consequence the SERS signal drops since the molecules are away from the highest field region. In this work, we show that dielectric nanoantennas overcome these limitations and allow having the same configuration for both SERS and SEF.

**Figure 5:**
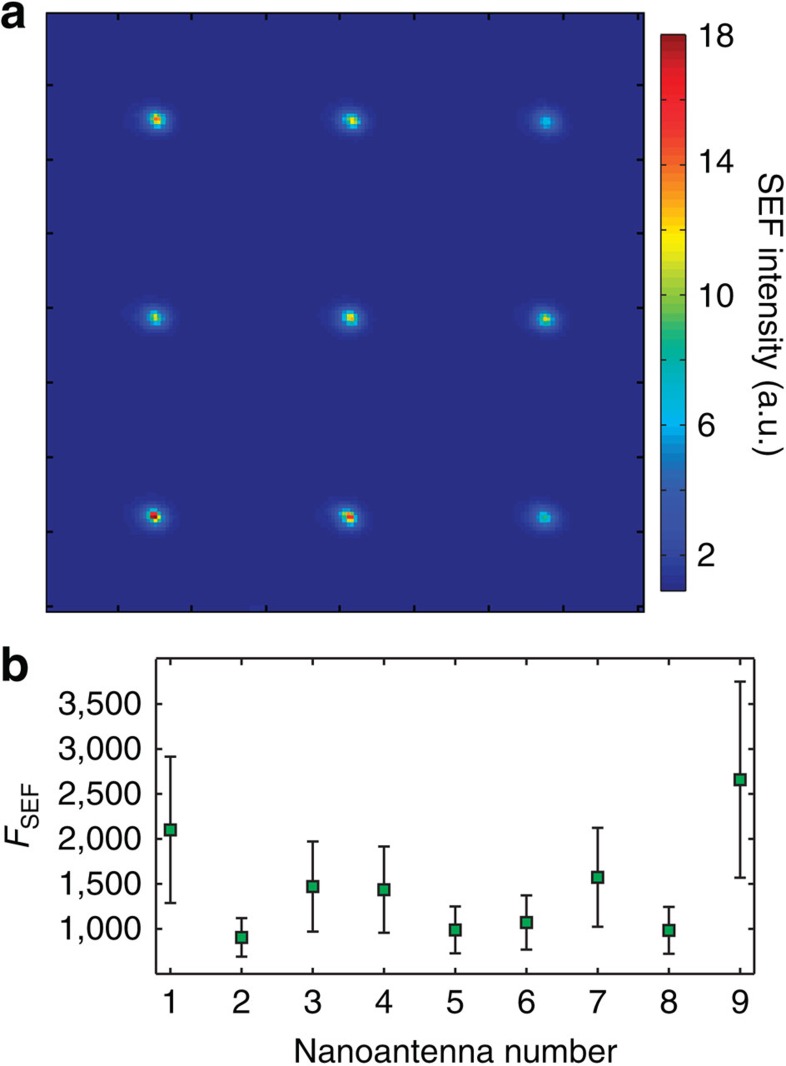
Surface enhanced fluorescence. (**a**) Experimental SEF map obtained for the Si antennas. It can be clearly seen that fluorescence is enhanced over the antennas. (**b**) SEF enhancement factor (*F*_SEF_) obtained from the maximum values over each antenna in **a**, calculated in similar way as *F*_SERS_. The error bars show half the difference between the minimum and the maximum value in each nanoantenna.

To carefully study this effect, an experimental SEF map was obtained (Fig. 5a) by normalizing the reference image for the Si case (Fig. 3b). A value of *F*_SEF_∼1,900 was obtained for the Si nanoantennas, as shown in Fig. 5b. This remarkable behaviour denotes an important difference between metallic and Si structures and opens the possibility of more elaborated SEF experiments with dielectric nanoantennas that provide a good SEF enhancement factor with no emission quenching and no spacers layers needed.

## Discussion

It is important to mention at this point that large Raman and fluorescence enhancement factors have been achieved through dielectric-based photonic resonators^48^,^49^. However, photonic crystal and whispering gallery mode devices optimized for these ends have characteristic sizes in the micrometre scale^50^. Although photonic crystals may be nanostructured, several ‘unit cells' are needed to build the artificial crystal to obtain the desired properties^51^,^52^, whereas for our Si-dimer nanoantenna all geometrical parameters are on the order of 100 nm. Moreover, while microcavity resonators use high Q-factors to generate field enhancements, Si nanoantennas use small modal volumes with low Q-factors. As such, the underlying physical phenomena are not the same. In fact, this difference gives rise to various advantages for our approach. High Q-factors imply a narrow spectral range of operation and difficulties associated with tuning the cavity to the material to be sensed. In contrast, small mode volume of the Si nanoantennas promotes Raman enhancement (due to field confinement) and fluorescence enhancement (due to the Purcell effect), while broadening the spectral range of utility as a result of the low Q-factor. Thus, small modal volumes allow light focusing in 3D nanoscale hot spots; also differing from one-dimensional field confinement recently shown in aluminium oxide nanosheets through Dyakonov surface waves^53^.

In conclusion, we have presented and experimentally demonstrated a novel type of Si-dimer nanoantenna exhibiting high near-field enhancement within a 20-nm gap at NIR wavelengths. These non-plasmonic nanoantennas are able to enhance the Raman scattering of a polymer thin film by a factor of ∼10^3^ and also allow surface enhanced fluorescence by a factor of ∼2 × 10^3^, avoiding the well-known fluorescence quenching effects observed for metallic structures when no spacer layers are used. Moreover, molecular thermometry measurements demonstrated that dielectric nanoantennas produce ultra-low heating when illuminating at their resonance wavelength, thus overcoming one of the main drawbacks of traditional plasmonic materials such as gold. Higher field enhancement factors which compare more favourably against the well-known metallic antennas are achievable by engineering future dielectric nanoantenna configurations. However, our claim here is that the scheme presented goes well beyond only the improvement of the field enhancement and, as so, our main point is that the plasmonic system suffers from limitations such as heating losses and non-radiative fluorescence quenching, amongst others, which are almost virtually absent in the dielectric case. Ultra-low heat conversion avoids reshaping or melting of the antenna thus important advances in many fields can be foreseen due to almost no restrictions in the power that can be delivered to these non-plasmonic nanoscale devices. For instance, dielectric-based nanoantennas can highly improve light-driven fields like nonlinear up-conversion processes, nanoelectronics and unperturbed sensing by enhanced spectroscopies or the study of nanoemitter behaviour.

We stress the fact that we have built a non-plasmonic nanoantenna with field enhancement capabilities of almost 50% of that of a gold-analogue system, but which exhibits a temperature increase more than one order of magnitude smaller, promotes fluorescence enhancement, and presents a hot-spot volume approximately five times larger than that of the Au system. This leads, for example, to 75% less heating in the Si nanoantennas compared with the Au plasmon analogue for the same Raman output signal. These results open new perspectives for the design, fabrication and applications of low-loss nanoantennas in the vis-NIR range. The possibility to have non-plasmonic materials that enhance the electromagnetic field without undesirable losses constitutes an important step in nanophotonics.

## Methods

### Numerical calculations

Nanoparticles composed of various materials (such as metals or semiconductors) can efficiently release heat under optical excitation. The heat-generation process involves not only absorption of incident photons, but also heat transfer from the nanoparticle to the surrounding matrix. The mechanism of heat release is very simple: the laser electric field strongly drives mobile carriers inside the material, and the energy gained by the carriers heats the material. Then, heat diffuses away from the nanostructure and leads to a temperature increase of the surrounding medium. Heat generation becomes especially strong in the case of metal nanoparticles in the regime of plasmon resonance. In the case of semiconductor nanoparticles, the heat-generation rate is much weaker since heat dissipation occurs through an interband absorption process with the creation of a single mobile electron and hole (exciton). In the absence of phase transformations, the temperature distribution around optically stimulated nanoparticles is described by the usual heat transfer equation:


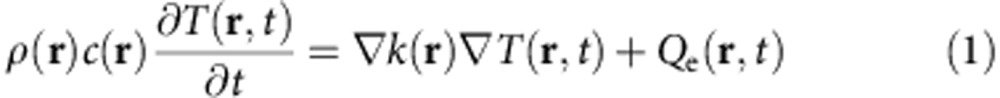


where *r* and *t* are the position and time, *T*(**r***,t*) is the local temperature and the material parameters *ρ*(**r**)*, c*(**r**) and *k*(**r**) are the mass density, specific heat and thermal conductivity, respectively. The function *Q*_e_(**r***,t*) represents the energy (heat) source coming from light dissipation (electromagnetic losses). The solution of this equation has a transient state, and after a characteristic time, it reaches its steady state^54^,^55^. Thermal processes in metals are fast, which means that a steady state is rapidly reached for typical incident powers and metal nanoparticle dimensions, similar to those used in nanomedicine^56^. To obtain the electromagnetic losses *Q*_e_(**r***,t*), a system of Maxwell's equations including appropriate boundary conditions must be written, which in the case of an ensemble of nanoparticles, such as a dimer, must be solved numerically. In our case, the whole process of light absorption and subsequent heat transfer between the nanostructure and the surrounding medium has been modelled by means of finite element simulations (in the same manner as in ref. 27). For easy implementation and reliability of the solution, we have chosen Comsol Multiphysics 4.3a, which provides state-of-the-art routines to solve partial differential equations. In our simulations, we have assumed the electromagnetic losses from the electromagnetic waves in the nanoparticles as the only heat source. Furthermore, we have assumed that the electromagnetic cycle time is short compared with the thermal time scale (adiabatic assumption). To take into account heat dissipation in our simulation region, we used a heat flux node across the outer boundaries, considering a heat transfer coefficient, dependent on the geometry and the ambient flow conditions. The heat transfer coefficient *h* can often be estimated by dividing the thermal conductivity of the convection fluid by a length scale^57^.

### Sample fabrication and preparation

Arrays of silicon dimers were fabricated by electron beam lithography on a backside polished silicon-on-insulator substrate, using the positive-tone electron-beam resist PMMA. First, the substrate was coated with PMMA and baked at ∼180 °C for 120 s. Then patterns of silicon dimers were defined by an electron beam exposure, followed by a development procedure. Subsequently, a 10 nm thick Cr film was deposited by thermal evaporation on the substrate, followed by lift-off. The structures were then transferred to the silicon substrates via a reactive ion etch using the Cr dimers as an etch mask and the buried oxide as an etch stop. The residual Cr was then removed via wet etching to obtain the Si dimers. Au antennas were fabricated through PMMA coating, electron-beam exposure, development, Au deposition and lift-off.

The samples were coated with a fluorescent dye in a polymer matrix to perform the temperature-mapping measurements. 950 PMMA A (MicroChem) in anisole was mixed with the fluorescent dye (Nile Red, Sigma Aldrich) to reach a final fluorophore concentration of 40 μM. This concentration was chosen after performing life-time measurements to find the upper-limit concentration before the onset of concentration quenching effects. The substrates were spin-coated at 3,500 r.p.m. for 1 min and then baked at ∼160 °C for 5 min. This procedure generates a homogeneous fluorescent layer of 200 nm in thickness. This last same procedure, but without the inclusion of the fluorophore, was performed to cover the sample with a PMMA film for SERS measurements.

### SERS mapping

A home-built inverted confocal microscope was used to obtain SERS images, using the appropriate filter set to allow efficient filtering for the excitation laser (from a tuneable CW Ti:Sapphire laser). All Raman images were taken over a 15 × 15 μm zone with a pixel size of 75 × 75 nm and a dwell time of 4 ms px^−1^. Raman spectra where taken with a home-made spectrometer consisting of a diffraction grating and a cooled CCD camera (see Supplementary Fig. 7 and Supplementary Fig. 8 for set-up schemes and Supplementary Note 7 for more details). SERS images were taken in resonance with the nanoantennas (860 nm), detecting a scattering band between 392 and 639 cm^−1^. The latter images were then normalized pixel by pixel with the scattering image taken in the detection wavelength range (890 nm), to account for the substrate contribution to obtain a normalized image *I*_R_. We then applied a Gaussian filter (with 5 × 5 px average and *σ*=1 px) to obtain Fig. 1f. The unfiltered normalized image was used to compute the maximum *I*_R_ in each nanoantenna (
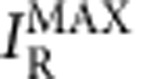
). The SERS enhancement factor was then calculated as:


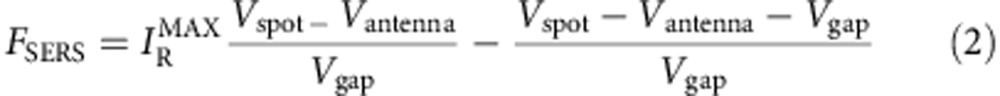


where *V*_spot_ was calculated with the full-width at half-maximum value for the laser spot; *V*_antenna_ and *V*_gap_ are the volumes of the antenna and the gap, respectively. Note that the dye coats everywhere, but not inside the antenna volume, and that is why *V*_antenna_ is subtracted in equation (2). Regarding the second term of this expression, it accounts for the contribution from outside the gap region. The gap was considered as a 20 × 20 nm × *h*_antenna_ rectangular box, where *h*_antenna_ stands for the nanoantenna height^40^,^37^. The obtained values for each nanostructure are shown in Fig. 1g, together with the theoretical expected value.

### Dual-beam confocal microscopy and imaging

The home-built confocal microscope also allows a dual-beam configuration. In this case the set-up consists of an inverted fluorescence confocal microscope, fed with a green low power imaging laser (*λ*=532 nm, ∼11 μW μm^−2^ at the sample) and a heating laser (CW tuneable Ti:Sapphire, with circular polarization at the sample). Both lasers are focused on the sample by a 40 × objective lens (NA=0.9) and the collected fluorescence is focused into a multimode optical fibre that acts as a confocal pinhole (see Supplementary Fig. 8 for set-up schematics and Supplementary Note 7 for details). The light coming out from the fibre is collimated and sent to an APD after filtering out the excitation light. Before each experiment, both lasers are carefully overlapped at the sample plane, to ensure that the heating and the fluorescence detection volumes fully coincide.

The fluorescence maps were taken over a 15 × 15 μm zone with a pixel size of 75 × 75 nm and a dwell time of 4 ms px^−1^. The background average value has been subtracted in each image for better displaying.

### Temperature calibration and thermal imaging

The calibration of fluorescence intensity versus temperature was done using a commercial fluorimeter (QuantaMaster 400, Quantum Technology International) with a temperature controller (TLC 50 Temperature Controlled Cuvette Holder for Fluorescence, Quantum Northwest). Emission spectra for Nile Red were recorded at different temperatures, as it is shown in Fig. 2c. To obtain the inset in the figure, we integrated the intensity in the detection range, 550–680 nm, and normalized it by the integrated intensity at ambient temperature (25 °C). These values were fitted with a third order polynomial function, which was used to transform intensity values into temperature.

The thermal imaging experiment consists of performing a series of images, starting with a bare fluorescence image taken as the reference. Further images in the same series, taken with one or both of the lasers alternatively, serves for bleaching correction, temperature determination and control of the preservation of the antennas. This procedure is repeated at every heating laser intensity, *I*_h_. Photobleaching effects were globally corrected by fitting a negative exponential function to the mean intensity of the images when chronologically ordered (see Supplementary Fig. 9 and Supplementary Note 8 for further details). The mean fluorescence intensity *I*_s_, on each nanoantenna was calculated by averaging the counts in a circular area with a radius of *r*_0_=5 px, centred at the nanostructure position. Similarly, the fluorescence intensity outside the nanoantennas, *I*_bg,_ was calculated. Calling *I*_s_,_0_ (*I*_bg,0_) the nanoantenna (background) intensity at the reference image and *I*_s,*lh*_ (*I*_bg,*lh*_) the nanoantenna (background) intensity with the laser heating on, the corrected intensity can be computed:


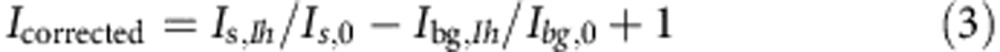


This intensity is then transformed into a temperature value with the calibration curve shown in the inset of Fig. 2c.

For the thermal mapping images, presented in Fig. 3, the mean intensity (computed in a region without nanoantennas) was subtracted to set the intensity value outside the dimers to zero. Negative counts then indicate zones where the fluorescence intensity is below the reference value. When heating on the nanoantenna the fluorescence emission of that area drops, as shown in the calibration curve—inset of Fig. 2c—and negative counts are computed in that region of the image.

## Additional information

**How to cite this article:** Caldarola, M. *et al.* Non-plasmonic nanoantennas for surface enhanced spectroscopies with ultra-low heat conversion. *Nat. Commun.* 6:7915 doi: 10.1038/ncomms8915 (2015).

## Supplementary Material

Supplementary Information Supplementary Figures 1-9, Supplementary Notes 1-8 and Supplementary References
