# Supplementary material for: Non-plasmonic nanoantennas for surface enhanced spectroscopies with ultra-low heat conversion
Source: Nat Commun. 2015 Aug 4;6:7915. doi: 10.1038/ncomms8915 (PMC4532885; doi:10.1038/ncomms8915)
Supplement: Supplementary Information — Supplementary Figures 1-9, Supplementary Notes 1-8 and Supplementary References [file ncomms8915-s1.pdf]

Supplementary information

Supplementary figures

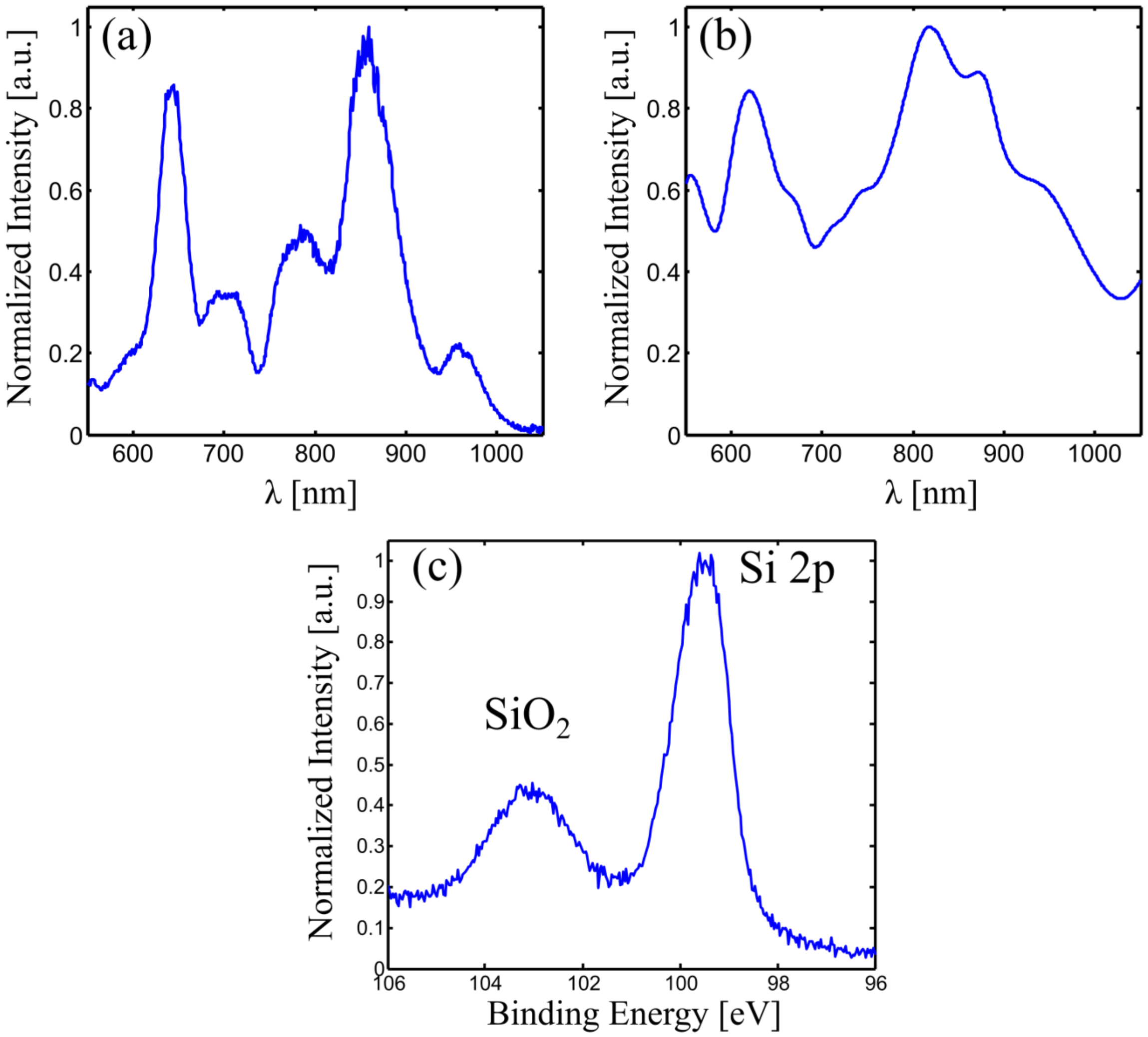

Supplementary Figure 1 | Silicon nanoantennas. (a) Experimental and (b) calculated dark-field spectra of the Si-dimer nanoantennas in air. (c) XPS data corresponding to the Si 2p region for the Si nanoantennas showing both contributions: pure Si (99.60 eV) and a small (<1 nm) Si oxide layer on-top (103.40 eV).

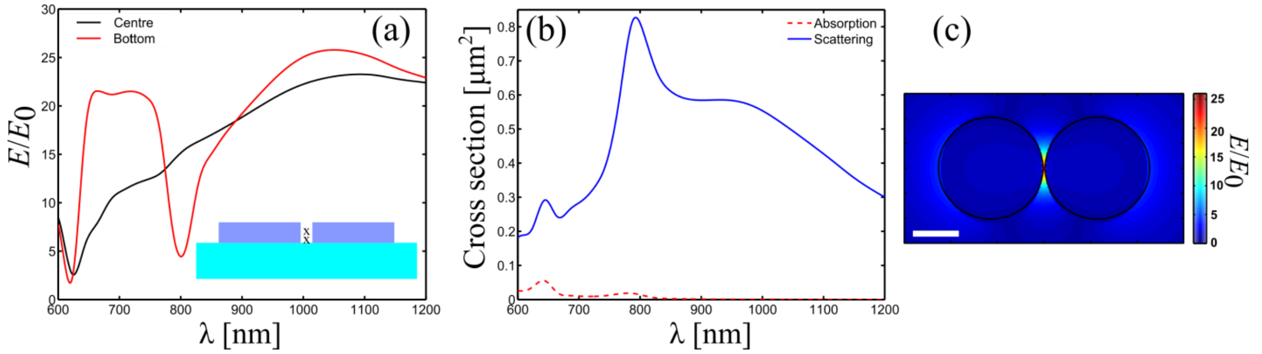

Supplementary Figure 2 | Si dimers with smaller gaps. Theoretical simulations for a dimer consisting of two identical Si disks with a diameter of 220 nm, a height of 200 nm, and a 4 nm gap in between. (a) Near-field enhancement as a function of the wavelength at different positions in the gap: centre (black line) and bottom (red line). These positions are schematically shown in the inset. (b) Absorption (red) and scattering (black) cross sections for the Si dimers with 4 nm gap size. (c) Near-field map distribution around the Si antennas with 4 nm gap at the same wavelength we performed our experiments (860 nm); E/E_0 can reach values over 22, thus $(E/E_0)^4$ being ~$2x10^5$.

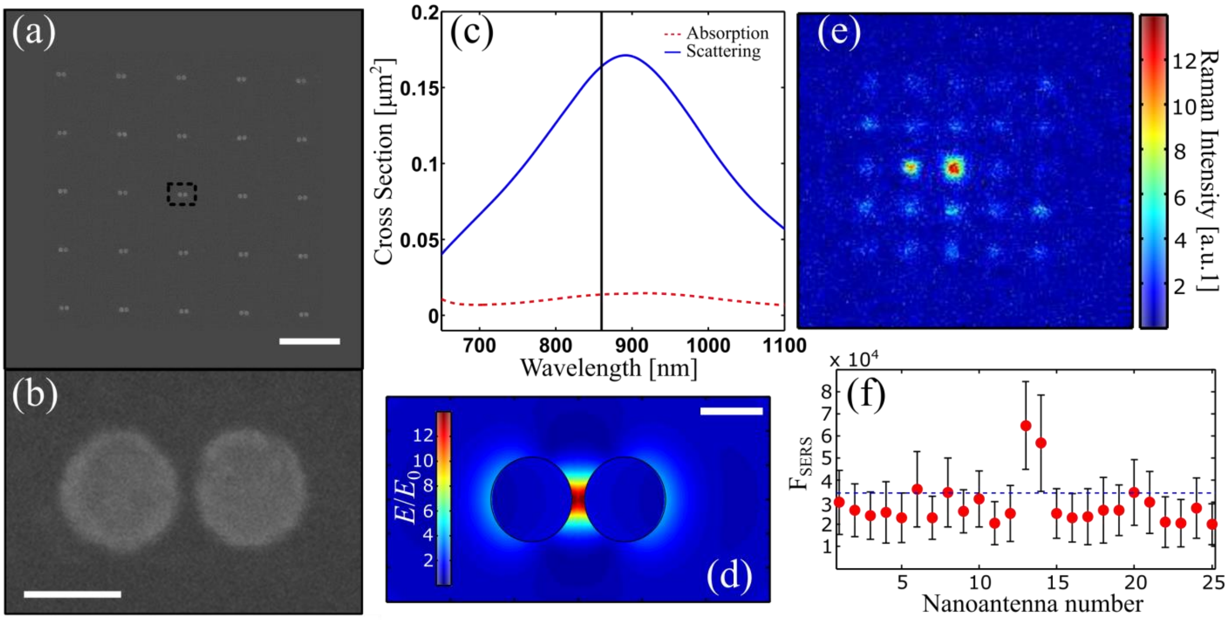

Supplementary Figure 3 | Gold nanoantennas. Design of dimeric Au disks nanoantennas and their enhancement performance. (a) SEM image of the nanostructures fabricated on a glass (SiO_2) substrate, which consist of two identical disks of 140 nm diameter and 40 nm height, with a 20 nm gap in between, disposed in a 5x5 array. Scale bar: 2 µm. (b) SEM image at higher magnification of a single nanoantenna, shown in the rectangle in (a). Scale bar: 100 nm. (c) Numerical calculation shows a scattering resonance near λ = 860nm (vertical black line) and a high absorption cross section. (d) Near field distribution map for the gold structure excited at resonance, showing good confinement of the electric field in the gap. Note that the maximum enhancement value is ~12. Scale bar: 100 nm. (e) Experimental 2D normalized Raman map, showing enhanced signal coming from the molecules close to the nanoantennas. (f) SERS enhancement factors obtained for each individual nanoantenna shown in (e). The dashed line corresponds to $(E_{max}/E_0)^4$ from (d) and the error bars show half the difference between the minimum and the maximum Raman intensity value in each nanoantenna.

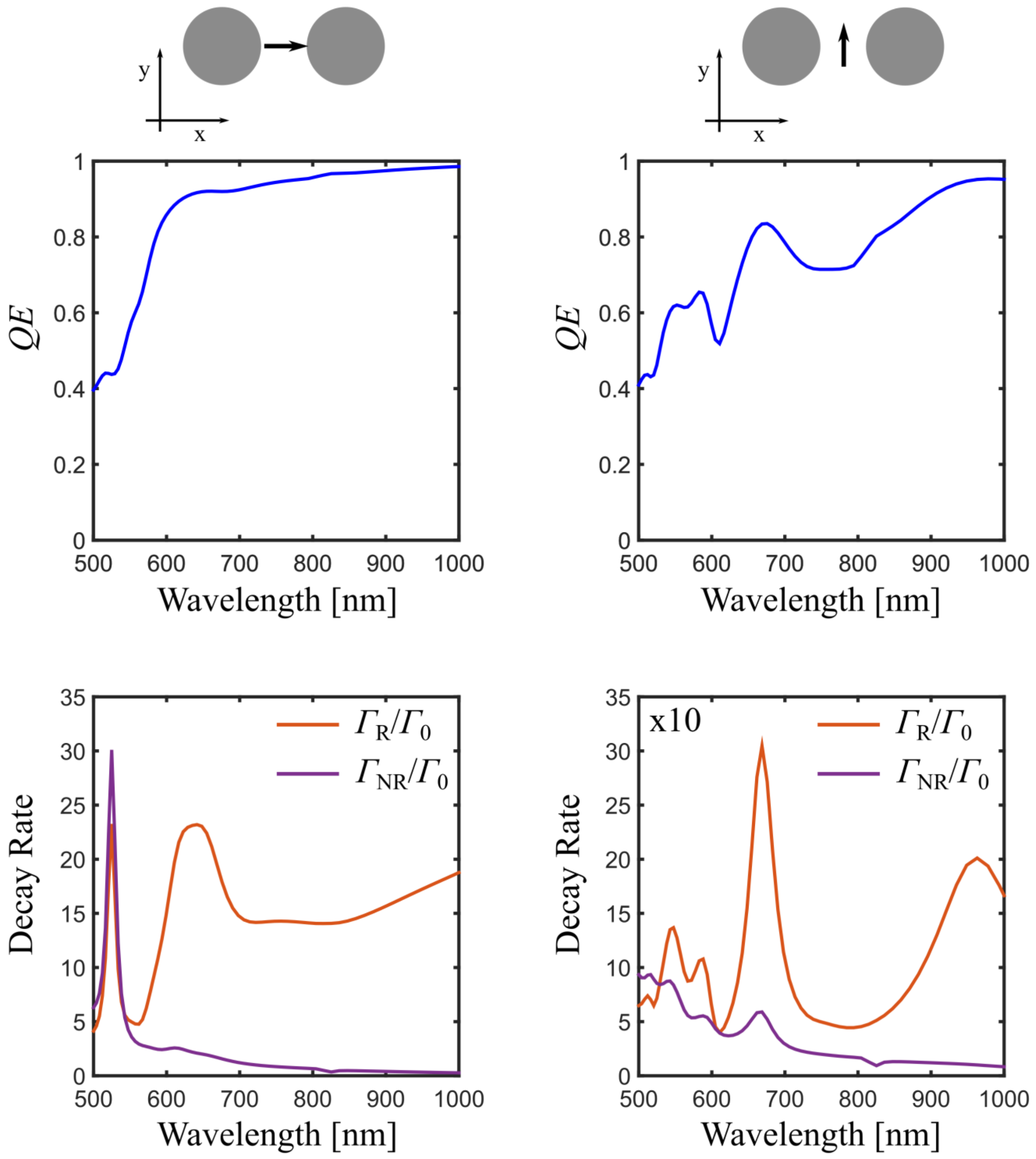

Supplementary Figure 4 | Dipole emission properties near Si nanoantennas. The orientation of the dipole relative to the dimer disk nanoantennas is shown schematically at the top of each column. In the Nile Red emission range (560-620 nm) the radiative rate is higher than the non-radiative rate. A scale factor is indicated in the figure when necessary for display.

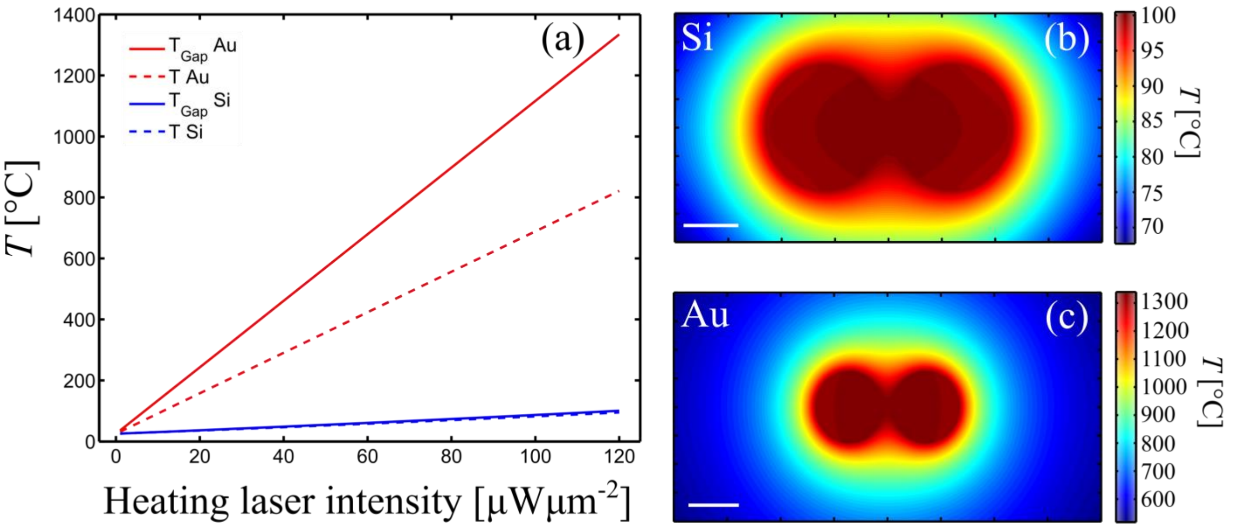

Supplementary Figure 5 | Temperature increase at the gap for high intensities. (a) The plots show the numerically calculated temperature increase for Au (red lines) and Si (blue lines) nanoantennas as a function of the incident laser intensity, polarized in the direction parallel to the dimers. The solid lines account for the temperature at the gap and the dashed curves for the mean temperature. (b)-(c) Calculated temperature map for I_h =120 mW μm^{-2} around the Si and Au disks, respectively. Scale bars: 100 nm.

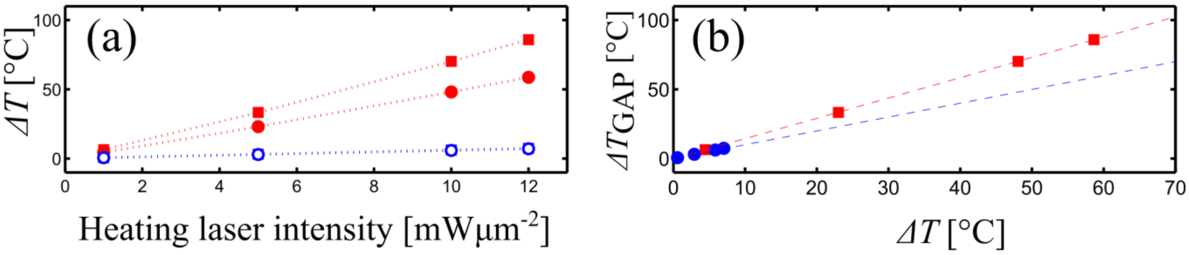

Supplementary Figure 6| Temperature numerical calculations. (a) Temperature increase predictions for Si (blue) and Au (red) nanoantennas, both in the gap and averaged in the detection volume as function of heating laser intensity. (b) Comparison between the temperature increase at the gap and the average temperature increase. The straight lines are fits to the data that were used to transform the measured temperature values into the gap temperatures in Figure 4(c) in the main text.

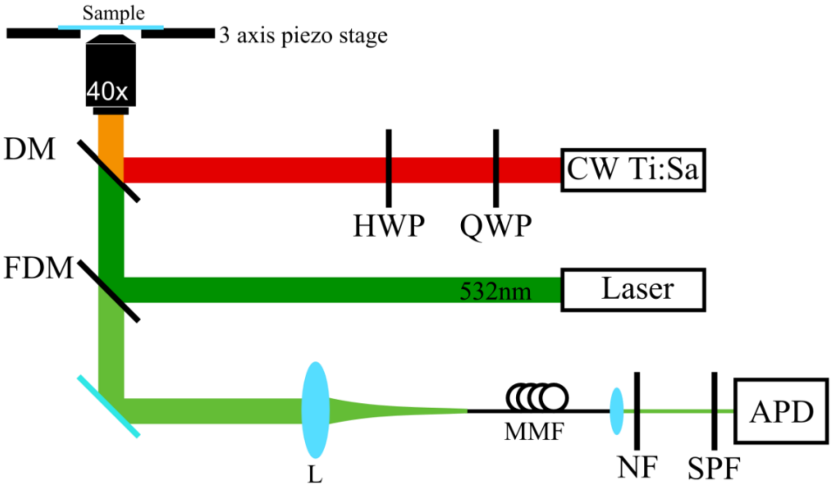

Supplementary Figure 7| Simplified scheme of the home-made dual beam fluorescence confocal microscope. The imaging (green) laser is sent to the sample using a fluorescence dichroic mirror (FDM) and mixed with the heating laser, a CW tuneable Titanium Sapphire (Ti:Sa) laser, at a second dichroic mirror (DM). A Half wave plate (HWP) and a quarter wave plate (QWP) are used to produce circular polarization at the sample. A 40x objective with high numerical aperture (NA = 0.9) was used to focus the lasers onto the sample and to collect the signal. Images were performed by raster scanning the sample using a 3 axis piezoelectric stage. The signals transmitted by the dichroic mirrors were then focused to a multimode optical fibre (MMF, 62.5 µm-core) that acts as a confocal pinhole, using a 30 cm achromatic doublet lens (L). After collimating the output of the MMF, the signal was filtered with a 532 nm notch filter (NF) and a short pass filter (SPF) at 680 nm, to ensure the complete removal of light from both the imaging and the heating lasers. Finally, the signal was recorded using an avalanche photodiode (APD).

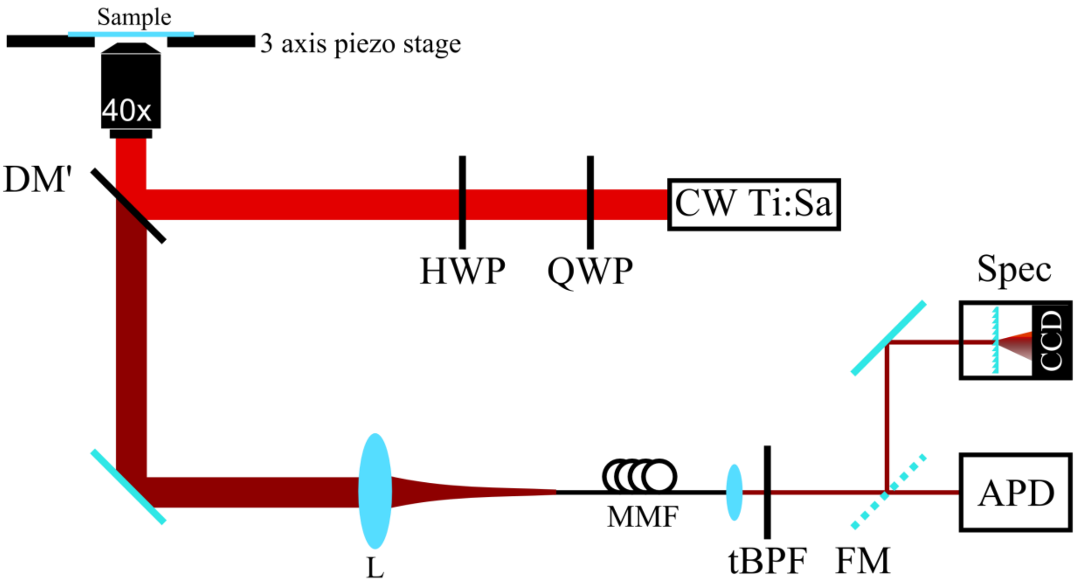

Supplementary Figure 8 | Simplified scheme of the home-made SERS microscope. The basic microscope is the same as in the fluorescence setup. In this case the Ti:Sa laser used for the excitation reflects in the DM' and focuses onto the sample. The Raman signal is collected and sent to the confocal fibre. A tuneable band pass filter (tBPF) is used to select the desired detection band. The position of the flipper mirror (FM) selects between the APD for imaging and the home-made spectrometer, based on a diffraction grating and a cooled CCD, for Raman spectra recording.

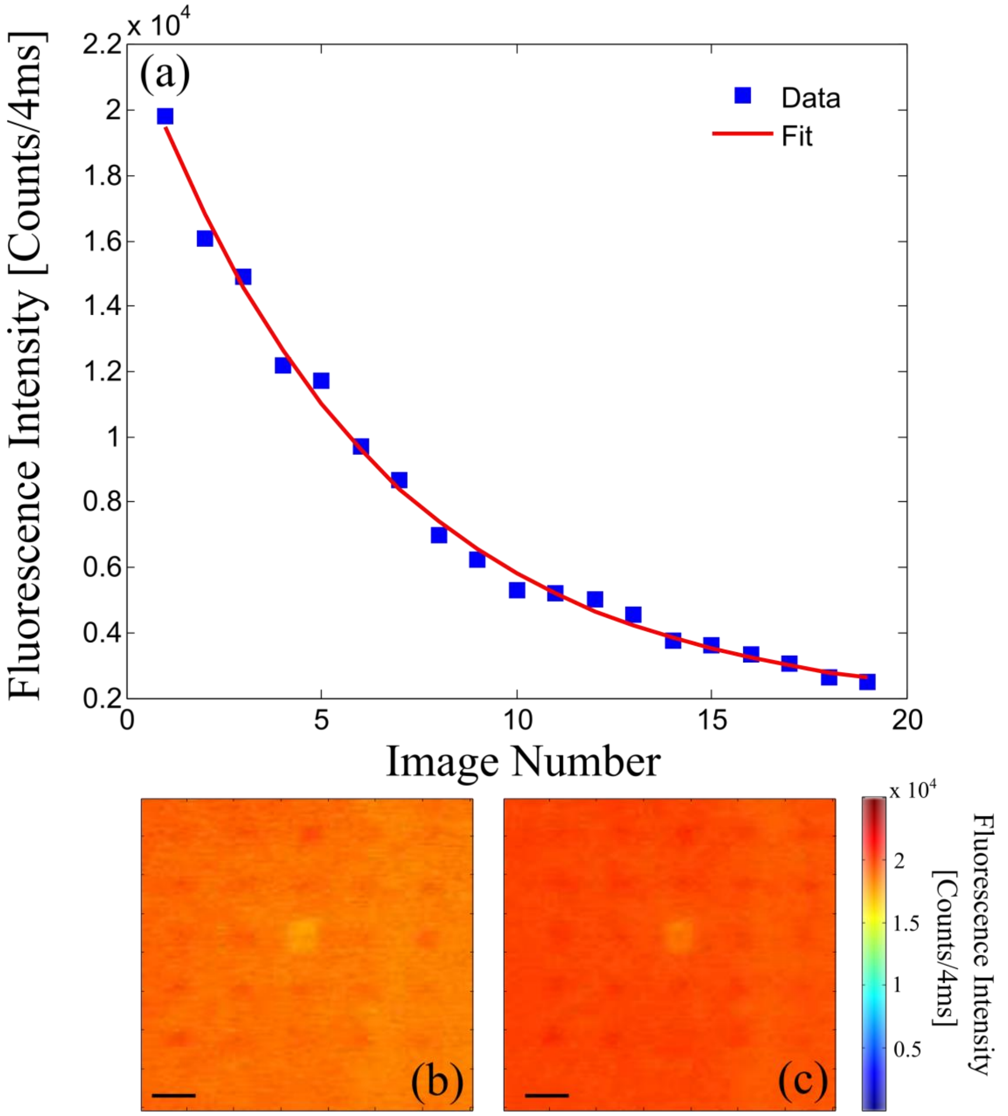

Supplementary Figure 9 | Photobleaching correction. (a) Photobleaching data and exponential fitting curve used to correct the intensity decrease generated by photoexposure. (b)-(c) Representative fluorescence images of the Au nanoantennas.

Supplementary notes

Supplementary note 1

Silicon nanoantennas: complementary characterization. Here we present further characterization (to the ones already shown in Figure 1 in the main text) for the Si nanoantennas. Firstly, we show in Supplementary Figure 1 the (a) measured and (b) calculated dark-field spectra for the Si dimers in air. As it can be seen, a very good agreement between both is achieved.

On the other hand, we also investigate the surface properties of our antennas in order to perform chemical modification of them. Traditional (metallic) plasmonic materials (i.e. Au, Ag, etc.) are easy to modify employing thiolated molecules that self-assembly on the surface of flat or nanostructured materials [1]. In order to explore possible alternatives to modify our Si-dimer antennas we performed XPS (X-ray photoelectron spectroscopy) measurements. XPS is a surface characterization technique and the penetration depth of X-rays in the material is generally on the order of 2 nm. Also, it is widely known that whenever silicon is exposed to air a < 1 nm layer of SiO_2 is formed [2]. Thus, we expect to be able to distinguish both the SiO_2 layer on top of the structures and the pure Si underneath. Indeed, as shown in Supplementary Figure 1(c), two peaks are clearly visible in the Si 2p region. The first peak at 99.60 eV corresponds to the pure Si underlying (Si $2p_{1/2}$ + Si $2p_{3/2}$) while the one at 103.40 eV has been assigned to the SiO_2 layer on top of the structures [3]. Thus, the ultrathin native oxide layer of the Si nanoantenna can be used to selectively bind a molecule to the SiO_2 surface. Silane molecules can be used to chemically modify (self-assembly) our nanoantennas in the same way as thiols are commonly used to modify metallic ones [4]. To corroborate this, we chemically functionalized the sample by using the (3-mercaptopropyl)-trimethoxy-silane (MPTMS) molecule, and characterized it also through XPS. As a result, the XPS spectrum showed presence of Si, SiO_2 and S, verifying that the functionalization was successful.

Supplementary note 2

Si dimers with smaller gaps. Our experimental results are limited solely by fabrication resolution. The showed experiments use 20 nm gap Si dimers, which to our knowledge is the smallest gap size ever achieved experimentally for these kind of structures [5]. However, theoretical simulations predict $(E/E_0)^4$ enhancements as large as ~$2x10^5$ in gaps as small as 4 nm for the same Si dimers and experimental conditions that we are using. Supplementary Figures 2 (a) and (b) shows the near field enhancement and the estimated absorption and scattering cross sections for a dimer consisting of two identical Si disks with a diameter of 220 nm, a height of 200 nm, and a 4 nm gap in between. Supplementary Figure 2(c) shows the near field map distribution at 860 nm for the previously mentioned Si dimers. This means that by only reducing the gap size from 20 nm to 4 nm, the E/E_0 factor can be increased 4 times. Moreover, for 4 nm gap size between Si spheres, the enhancement $(E/E_0)^4$ can reach values over ~10^6 [6]. This would allow in principle single-molecule SERS detection by using non-plasmonic nanoantennas [7].

Supplementary note 3

Gold nanoantennas design and characterization. In order to compare the thermal behaviour of the presented silicon nanoantennas, gold nanoantennas of the same shape but different sizes were fabricated as described in the Methods section. The gold disks are organized in a 5x5 array, shown in Supplementary Figure 3(a), and have 140 nm diameter, 40 nm height and 20 nm gaps, as shown in Supplementary Figure 3(b). These dimensions were selected to achieve a dipolar resonance in the same spectral region than the silicon antennas presented in the main paper. Supplementary Figures 3 (c) and (d) show the scattering and absorption spectra and the near-field map, respectively. The resonance centred at 860 nm is a dipolar resonance which provides a field enhancement (E/E_0) of ~12.

SERS imaging was performed over these antennas using the exact same conditions as for the silicon antennas, as can be seen in Supplementary Figure 3(e). From this image we calculated the experimental values for F_{SERS}, alike the silicon case, which showed an excellent agreement with the expected value from numerical simulations, as it can be seen in Supplementary Figure 3(f).

Supplementary note 4

Ideal dipole emission spectra and SEF. Dielectric antennas present a structure with unique properties, allowing them to enhance the decay rates of single emitters while producing low-losses and heating of the local environment in comparison with metallic antennas [6,10]. The decay rate of a dipolar emitter placed in the gap is modified due to two competitive processes: transfer of the energy from the emitter to the nanoparticle (observed exclusively for lossy nanoparticles) [11,12] and the coupling of the field originated by the emitter to the outgoing radiation [13,14]. The resulting modification of the emitter's decay rate, known as the Purcell effect, has been extensively studied and applied to designing efficient nanoantennas for single photon emission [15], enhancement of the fluorescence intensity [16,17] and emission directivity [14].

To model the dipolar transition moment of a fluorescent molecule, we treat our emitter as an ideal electric dipole (same as in ref. [6]), with an intrinsic quantum efficiency of 1 and a decay rate Γ_0. The dipole is placed in the geometric centre of the Si dimer and both the power radiated by the system and the dissipation of the energy from the emitter via ohmic losses are calculated. These quantities, when normalized by the power emitted in the absence of the antenna, yield the enhancement of the emitter's radiative decay rate, Γ_R/Γ_0, and of the non-radiative processes, Γ_{NR}/Γ_0. The quantum efficiency is then defined as $QE = \Gamma_R/(\Gamma_R + \Gamma_{NR})$. The cases of an emitter oriented either along or perpendicular to the antenna symmetry axis are both considered and a comparison of the radiative and non-radiative decay rate enhancement spectra, together with the resulting quantum efficiencies, are shown in Supplementary Figure 4.

These calculations show that there is a notable radiative enhancement in the spectral zone that overlaps with the Nile Red emission spectra while the non-radiative decay rate is negligible in that spectral range. Thus, the combination of low-losses and enhancement obtained in the dielectric nanoantennas makes them beneficial for modifying and controlling the emission of dipole emitters.

Supplementary note 5

Temperature increase at high intensities. Numerical calculations show that for high excitation intensity values, the temperature in gold nanoantennas increases dramatically (reaching values over 1300 °C), which will certainly damage the structures [18]. On the other hand, for these high intensities, the temperature in Si nanoantennas does not exceed 100 °C, as shown in Supplementary Figure 5(a). This allows the possibility of working with Si structures at much higher excitation intensities, where metallic structures cannot be used. The spatial distribution of the temperature around the Si nanoantennas for high heating-laser intensities (120 mW μm^{-2}) is shown in Supplementary Figure 5(b).

Contrary to the SERS effect, for which the signal scales with $(E/E_0)^4$, in standard Raman spectroscopy the scattering intensity is linear with the incident power. Increasing the laser power by a factor of 5 (at a wavelength off resonance) will not produce the same increase of 10^3 as would be seen with a field enhancement factor of 5.5 working at resonance ($1000^{1/4} = 5.6$). Rather, it will only generate a mere 5-fold Raman signal amplification [19].

In our experiment, we note that by increasing less than 5 times the incident power for the Si nanoantennas we can get the same SERS output signal than the Au-analogue dimer but with 75% less heating. For example, at 120 mW μm^{-2} incident power the Si nanoantenna reaches a temperature of 100 °C, with an associated SERS signal proportional to $(120*5.5*5.5)^2 = 13x10^6$, equivalent to 25 mW μm^{-2} for the Au-analogue system $(25*12*12)^2 = 13x10^6$, which would heat-up to about 325 °C. Then, for the first case the temperature increase is 75 °C (room temperature is 25 °C) while for the second the corresponding value is 300 °C, which gives a ratio of 25% (Si/Au). This fact could be further improved by working with GaP antennas for example, where absorption in the visible is reduced compared to Si [10].

Supplementary note 6

T_{GAP} vs T. Here we discuss numerical calculation results for the thermal behaviour of the silicon and gold nanoantennas. The temperature increase at the gap and the average temperature increase (in the confocal detection volume) values for different heating laser intensities are shown in Supplementary Figure 6 (left column), for λ = 860nm. In Supplementary Figure 6 (right column) we show the relationship between ΔT_{GAP} and ΔT, from which we extracted a proportionality factor of 1.46 (1.05) for gold (silicon) at λ = 860nm. These values were used to build Figure 4(c) in the main text.

As it can be noticed, the slopes of T_{GAP} vs. T for Si and Au are different. This variation emerges as a consequence of the different dimensions between the dielectric and metallic disks, which are 220 nm (140 nm) diameter and 200 nm (40 nm) height for Si (Au). Since for the former the proportion of molecules in the gap (with respect to the total amount of molecules sensed) is greater than for the metallic case, a lower $\Delta T_{GAP}/\Delta T$ ratio is achieved.

Supplementary note 7

Experimental setup. The experimental setup consists of a home-made confocal microscope with an additional laser beam used as the heating laser. This microscope was set for Nile Red fluorescence measurements and then slightly modified for SERS imaging and Raman spectra measurements.

Supplementary Figure 7 shows schematically the setup for the fluorescence measurements. The imaging laser (λ=532nm, Coherent Compass 315M) is reflected in a fluorescence dichroic mirror (FDM, Di02-R532-25x36, Semrock) and sent to the 40x objective lens (NA = 0.9 Olympus UPLANSAPO). The heating laser, a CW Ti:Sapphire laser (Model MTS, KMLabs), is mixed with the green laser in a second dichroic mirror (DM 750dcspxr, Chroma Technology corp.) and overlapped in the sample plane with the imaging laser at the sample. A half wave plate (Thorlabs) and a quarter wave plate (Thorlabs) are used to get circular polarization at the sample. The sample is raster scanned over the focus spot by a 3 axis piezo stage (PI 517.3cl, Physik Instrumente GmbH & Co) to get a confocal image. The emitted fluorescence from Nile Red (emission peak at 570 nm) is collected by the same objective and transmitted through both dichroic mirrors and focused with an achromatic doublet into a multimode fibre (MMF) with 62.5 µm-core (M31L01, Thorlabs). The output of the fibre is then collimated (F220FC-A, Thorlabs) and the imaging laser is filtered with a notch filter (NF, NF03-532E-25, Semrock) while the heating laser is filtered with a short pass filter (SPF, ET680sp2p, Semrock). This filtering combination allows a detection range between 550 and 680 nm. Finally, the detector is an avalanche photodiode (APD, Perkin Elmer SPCM-AQR-13).

For SERS imaging, the setup is shown schematically in Supplementary Figure 8. The main features coincide with the fluorescence setup. The main changes consist on the modification of the spectral range for detection and the use of the Ti:Sa laser as unique excitation laser. The dichroic mirror (DM') reflects the laser at 860 nm and transmits the emitted Raman stokes signal, at longer wavelengths (DM FF875-D101-25X36, Semrock). The Raman scattering signal is sent to the confocal fibre as it is done for fluorescence. After collimation a tuneable band pass filter (tBPS, TB901-900/11-25X36, Semrock) is used to select the desired detection band (890-910 nm).

Contrary to SERS measurements in Si nanoparticles solutions [20], in our case we want to image and quantify the SERS enhancement factor for every single Si-dimer nanoantenna [13]. For this reason, a flipper mirror was added after collimation to allow selection between two different paths: one for imaging purposes and other for spectra recording. In the imaging case, the detector is the APD and for Raman spectra recording is a home-made spectrometer based on a diffraction grating (Thorlabs) and a cooled CCD (altaU2000, Apogee Imaging Systems Inc).

Supplementary note 8

Photobleaching corrections for the thermal imaging process. In Methods section of the main paper (see Temperature calibration and thermal imaging) we do a detailed description of the calibrations performed to compute the temperature and perform the thermal imaging.

Here we discuss the calibration curve/images used to account for the photobleaching of the dye along the thermal mapping/imaging process. Each fluorescence image was corrected by a factor extracted from measuring the mean intensity vs. photoexposure time (which is proportional to the image number) in a zone without nanoantennas. An exponential decay function was used to fit the data. In Supplementary Figure 9 we show this curve and the fitting used for correction, together with two consecutive images. Additionally, we verified that the intensity profile was unaltered when analysing regions including dimers. We note that within the experimental conditions employed, photobleaching produced less than a 10% decrease of the intensity from image to image. In Supplementary Figure 9 we show a representative curve and two representative images of the Au nanoantenna sample. The R^2 factor of the exponential fit was found to be >99%.

Supplementary References

[1] Vericat, C., et al. Self-assembled monolayers of thiolates on metals: a review article on sulfur-metal chemistry and surface structures. *RSC Advances*, **4**(53), 27730-27754(2014).

[2] Morita, M., Ohmi, T., Hasegawa, E., Kawakami, M., & Ohwada, M. Growth of native oxide on a silicon surface. *Journal of Applied Physics*, **68**(3), 1272-1281 (1990).

[3] Bashouti, M. Y., Sardashti, K., Ristein, J., & Christiansen, S. H. Early stages of oxide growth in H-terminated silicon nanowires: determination of kinetic behavior and activation energy. Physical Chemistry Chemical Physics, **14** (34), 11877-11881 (2012).

[4] Haensch, C., Hoeppener, S., & Schubert, U. S. Chemical modification of self-assembled silane based monolayers by surface reactions. *Chemical Society Reviews*, **39** (6), 2323-2334 (2010).

[5] Bakker, R. M., et al. Magnetic and electric hotspots with silicon nanodimers. *Nano Letters*, **15** (3), 2137-2142 (2015).

[6] Albella, P., Poyli, M. A., Schmidt, M. K., Maier, S. A., Moreno, F., Sáenz, J. J., & Aizpurua, J. Low-loss Electric and Magnetic Field-Enhanced Spectroscopy with subwavelength silicon dimers. *The Journal of Physical Chemistry* C, **117** (26), 13573-13584 (2013).

[7] Le Ru, E. C., & Etchegoin, P. G. (2012). Single-molecule surface-enhanced Raman spectroscopy. Annual review of physical chemistry, 63, 65-87.[8] Dodson, S., Haggui, M., Bachelot, R., Plain, J., Li, S., & Xiong, Q. Optimizing electromagnetic hotspots in plasmonic bowtie nanoantenna. *The Journal of Physical Chemistry Letters*, **4** (3), 496-501 (2013).

[9] Heeg, S., et al.Plasmon-enhanced Raman scattering by carbon nanotubes optically coupled with near-field cavities. *Nano letters*, **14** (4), 1762-1768 (2014).

[10] Albella P., Alcaraz de la Osa R., Moreno F., Maier S. A. Electric and Magnetic Field Enhancement with Ultralow Heat Radiation Dielectric Nanoantennas: Considerations for Surface-Enhanced Spectroscopies. *ACS Photonics,* **1**(6), 524–529 (2014).

[11] Anger, P., Bharadwaj, P., & Novotny, L. Enhancement and quenching of single-molecule fluorescence. *Physical review letters*, **96** (11), 113002 (2006).

[12] Mackowski, S., et. al. Metal-enhanced fluorescence of chlorophylls in single light-harvesting complexes. *Nano letters*, **8**(2), 558-564 (2008).

[13] Ruppin, R. Decay of an excited molecule near a small metal sphere. *The Journal of Chemical Physics*, **76**(4), 1681-1684 (1982).

[14] Curto, A. G., Volpe, G., Taminiau, T. H., Kreuzer, M. P., Quidant, R., & van Hulst, N. F. Unidirectional emission of a quantum dot coupled to a nanoantenna. *Science*, **329**(5994), 930-933 (2010).

[15] Novotny, L., & Van Hulst, N. Antennas for light. *Nature Photonics*, **5**(2), 83-90 (2011).

[16] Laroche, M., Albaladejo, S., Carminati, R., & Sáenz, J. J. Optical resonances in one-dimensional dielectric nanorod arrays: field-induced fluorescence enhancement. *Optics letters*, **32**(18), 2762-2764 (2007).

[17] Aroca, R. F., Teo, G. Y., Mohan, H., Guerrero, A. R., Albella, P., & Moreno, F. Plasmon-enhanced fluorescence and spectral modification in SHINEF.*The Journal of Physical Chemistry C*, **115**(42), 20419-20424 (2011).

[18] Kuhlicke, A., Schietinger, S., Matyssek, C., Busch, K., & Benson, O. In situ observation of plasmon tuning in a single gold nanoparticle during controlled melting. *Nano letters*, **13**(5), 2041–6 (2013).

[19] Le Ru E., Etchegoin P., Principles of surface enhanced Raman spectroscopy and related plasmonic effects, Elsevier, 2009

[20] Rodriguez, I., Shi, L., Lu, X., Korgel, B. A., Alvarez-Puebla, R. A., & Meseguer, F. Silicon nanoparticles as Raman scattering enhancers. *Nanoscale*, **6** (11), 5666-5670 (2014).
